# Agitated Depression Associated With Flurazepam Discontinuation

**DOI:** 10.1155/2024/8845349

**Published:** 2024-09-20

**Authors:** Mohamed Salih, Reem Mohamed Osman, Wala Alim, Leena Khalid, Wafa Sosal, Danya Ibrahim, Yassir Mahgoub

**Affiliations:** ^1^ St Davne'ts Campus Cavan Monaghan Mental Health Service, Rooskey, Monaghan, Ireland; ^2^ Faculty of Medicine Al-Neelain University, Khartoum, Sudan; ^3^ Clinical Documentation Improvement Specialist Orange Global Medical Centre, Corona, California, USA; ^4^ Faculty of Medicine University of Khartoum, Khartoum, Sudan; ^5^ Psychiatry and Behavioral Health Penn State College of Medicine, Hershey 17033, Pennsylvania, USA

**Keywords:** agitated depression, antidepressants, GABA agonists, melancholia

## Abstract

Agitated depression, also known as melancholia agitata, is a variant of depression characterized by severe symptoms of psychomotor agitation, inner unrest, anxiety, restlessness, prominent vegetative symptoms, and a high risk of suicide. This form of depression is reported to worsen with antidepressants and potentially improve with the use of ECT, lithium, antiepileptics, antipsychotics, and benzodiazepines. We describe a case of a 73-year-old female with a prior history of depression and generalized anxiety disorder who was maintained on flurazepam for 44 years and was admitted for severe depression with psychomotor agitation, prominent vegetative symptoms, thought perseveration, indecisiveness, and psychotic features that emerged following the discontinuation of flurazepam. Symptoms did not resolve with the use of alternative benzodiazepines such as nitrazepam and temazepam and further worsened with the use of several antidepressants. She finally had a complete resolution of these symptoms with a combination of alprazolam, zopiclone, and olanzapine. This case provides insight into this unique variant of depression and the role of GABA agonists in its pathology and management.

## 1. Introduction

Agitated depression, also known as melancholia agitata, is a variant of depression characterized by severe symptoms of psychomotor agitation, inner unrest, anxiety, restlessness, prominent vegetative symptoms, and a high risk of suicide [1]. Mahgoub et al. [2] expanded the definition of agitated depression under the term melancholia to include alternating states of psychomotor agitation or retardation, prominent vegetative symptoms such as weight loss and insomnia, thought perseveration, and indecisiveness. Both Koukopolous et al. [1] and Mahgoub et al. [2] suggested that this variant can worsen with the use of classic antidepressants and improve with options like electroconvulsive treatment (ECT), lithium, antipsychotics, and gamma-aminobutyric acid (GABA) agonists [1, 2].

We describe a case of a 73-year-old female with a prior history of depression and generalized anxiety disorder who was maintained on flurazepam for 44 years and was admitted for severe depression with psychomotor agitation, prominent vegetative symptoms, thought perseveration, indecisiveness, and psychotic features that emerged following the discontinuation of flurazepam. Symptoms did not resolve with the use of alternative benzodiazepines such as nitrazepam and temazepam and further worsened with the use of several antidepressants. She finally had a rapid and complete resolution of these symptoms on a combination of alprazolam, zopiclone, and olanzapine. This case provides insight into this unique variant of depression and the role of GABA agonists in its pathology and management.

## 2. Case Presentation

A 73-year-old female with a medical history of breast cancer, in complete remission, hypothyroidism, and hypertension, was maintained on levothyroxine 75 *μ*g and ramipril 5 mg once daily. She also had a remote history of generalized anxiety disorder, which was diagnosed 44 years before her hospitalization and maintained on flurazepam 60 mg daily with complete remission of symptoms. There were no concerns about benzodiazepine dependence and no prior history of withdrawal or problematic use of benzodiazepines. She was diagnosed with major depression 26 years before her current presentation and has been on escitalopram 15 mg daily for the last 10 years until she had a recurrence of symptoms of depression 5 months before her hospitalization. These depressive symptoms emerged following the sudden discontinuation of flurazepam, which occurred secondary to the national shortage in Ireland in 2023.

The symptoms of worsening depression occurred on the fifth day following the discontinuation of flurazepam, where she reported pervasive and persistent lowering of mood, low motivation, anhedonia, worsening of attention and memory, and poor sleep, where she struggled with falling asleep and staying asleep. Her appetite became poor, with excessive anxiety, increased restlessness, and excessive rumination over having a ringing sensation in her ears. No tremors or confusion occurred. These symptoms emerged almost 21 weeks before her hospitalization. Due to concerns about benzodiazepine withdrawal following flurazepam discontinuation, her general practitioner prescribed her nitrazepam 5 mg once daily, with no improvement reported. Hence, it was later replaced by temazepam 10 mg once daily 1 week later. This regimen was used for 1 month with a lack of improvement, and her previous symptoms continued to worsen, especially her poor sleep, appetite, and weight loss, where she lost a total of 12 kg. Hence, she was subsequently tried on mirtazapine 15 mg daily. However, her symptoms of depression worsened, and she became more restless, irritable, and intense when seen after 3 weeks of using mirtazapine. She had notable ambivalence about seeking help, leading to a suicidal attempt by overdose, where she swallowed 20 pills of an older prescription of fluoxetine 20 mg. She required medical hospitalization, where escitalopram 15, alprazolam 0.25 mg twice daily, and temazepam 20 mg daily were added to the previous mirtazapine 15 mg daily 1 week later.

Despite the changes, she continued to struggle with previous symptoms of depression in addition to the emergence of passive death wishes, significant indecisiveness about treatment, and excessive rumination over potential weight gain side effects of mirtazapine despite not gaining weight, and ultimately, she discontinued mirtazapine. Hence, escitalopram was also discontinued after being used for 2 months, and she was trialed on venlafaxine instead, which was started at a dose of 37.5 mg daily and was increased to 75 mg daily after 1 week, with no improvement. Temazepam was temporarily discontinued for 10 days due to market unavailability. During that time, she was trialed on zopiclone 7.5 mg daily with no improvement in insomnia, depression, or anxiety, and zopiclone was discontinued once temazepam was became available in the market.

Due to the persistence of symptoms and the concerns about the excessive use of benzodiazepines, alprazolam was discontinued 3 weeks before her admission. This change was followed by the worsening of symptoms of depression, increased anxiety, irritability, increased restlessness, poor appetite, and poor sleep, in addition to the emergence of delusions about her family plotting against her and the delusions about her neighbors commenting on her foul smell. Due to the persistence of symptoms and the extreme ambivalence about seeking help, she was hospitalized involuntarily. Her physical examination and medical investigations were unremarkable. Both alprazolam 0.25 mg twice daily and zopiclone 15 mg were resumed to the previous regimen of venlafaxine 75 mg daily. In addition, olanzapine was started and titrated to 10 mg daily over 12 days. With the above combination, her prior symptoms of depression, anxiety, irritability, indecisiveness, rumination, insomnia, poor sleep, poor appetite, and psychosis improved. She was discharged 20 days after hospitalization, and her symptoms remained in remission for 5 months after discharge.

## 3. Discussion

Richarz [3] coined the term melancholia agitans. He distinguished between the nature of “racing thoughts” associated with melancholia and the flight of ideas seen in mania. However, the term “agitated depression” was first introduced to literature by Weygandt [4] as a mixed manic depressive state. Kraepelin [5], the first scholar to describe this mixed affective state systematically, coined the term “manic depressive insanity.” Subsequently, Kraepelin [5] developed a theoretical framework with three psychological domains—thoughts, affection, and psychomotor volition—to describe the various states of mixed mood disorders. He suggested that different mixed mood disorders were caused by the unsynchronized stimulation and inhibition in these three domains. His description of “excited depression” and “depression with a flight of ideas” seems to be closely related to agitated depression, where the train of thought is inhibited or excited, respectively [5]. Later scholars viewed melancholia agitata as a form of mixed manic depressive insanity [6–8], while others considered it as a type of anxiety psychosis [9].

Koukopoulos et al. proposed that agitated depression or melancholia agitata is a form of depression that is characterized by an inner agitation with at least three of the following symptoms must be present: (i) racing or crowded thoughts, (ii) irritability or unprovoked feelings of rage, (iii) absence of signs of retardation, (iv) talkativeness, (v) dramatic descriptions of suffering or frequent spells of weeping, (vi) mood lability and marked emotional reactivity, and (viii) early insomnia. They suggested the use of ECT as a first-line option. In addition, they suggested the use of antipsychotics, antiepileptics, lithium, and benzodiazepines as alternative options. They highlighted the problematic effect of antidepressants on this variant of depression, as they can worsen symptoms [1]. These criteria were proposed for the DSM-5, but the task force elected not to recognize it as a variant of depressive disorder. While an argument can be made that the agitated depression variant can be captured under the mixed mood specifier, the current requirement is to fulfill the mixed mood specifier mandates meeting criteria for a major depressive episode and at least three of the manic symptoms. However, DSM-5 excluded overlapping mood criteria that occur in both depressive and manic episodes, such as agitation, irritability, and mood lability. While this was done to improve specificity, these overlapping criteria are the core features of agitated depression, as suggested by Koukopoulos et al. [1]. This made the DSM-5 mixed criteria almost unable to recognize or capture agitated depression [10].

Recently, Mahgoub et al. [2] proposed criteria for melancholia that described overlapping features of agitated depression described by Koukopolus [1] but also included an alternating state of psychomotor retardation, indecisiveness, and perseveration. They suggested that this variant of depression might worsen with the use of antidepressants, and they might respond to GABA agonists such as lorazepam and zolpidem in a rapid on-and-off manner. This is in contrast to the current DSM-5-TR criteria for melancholia, which is considered a specifier for major depression and bipolar disorders and is characterized by anhedonia and/or lack of reactivity, with at least three of the following: excessive delusional guilt, significant anorexia or weight loss, early-morning awakening, depression worse in the morning, empty mood, and marked psychomotor agitation or retardation [11].

GABA is the brain's principal neurotransmitter mediating neural inhibition. The most compelling evidence suggests that low GABA levels in plasma, cerebrospinal fluid, or removed cortical tissue could play a role in depression disorders directly or indirectly [12]. Furthermore, GABA deficits appear to be significant in melancholic and treatment-resistant subtypes of depression [13–15], while reductions in depressed patients not meeting the criteria of melancholia and in bipolar patients were less [12, 15–17].

Flurazepam is a benzodiazepine used as a sleeping aid [18]. By binding to the GABA-A receptor, benzodiazepines can increase the inhibition of synaptic transmission mediated by GABA. Following the introduction of flurazepam, several benzodiazepines and analogs with shorter half-lives and better tolerance were introduced, and flurazepam became fashionable.

The availability and use of benzodiazepines in Ireland and several European countries have changed over the last few years, ultimately resulting in a significant reduction in the market supply for various benzodiazepines. This issue was precipitated by recent regulations in several European countries to control the prescription and use of benzodiazepines and various controlled medications that were introduced in Ireland in 2017 [19–22]. In addition, during the COVID-19 pandemic, several people reported worsening anxiety and depressive symptoms, potentially increasing the demand for these medications [23]. A study conducted in the USA showed a significant increase in the description and the use of benzodiazepines around COVID-19 pandemic [24]. In addition, the supply chain of various medications was interrupted by a reduced workforce and supply of precursor chemicals [25]. All the above created the perfect storm that resulted in the disruption of the use of various benzodiazepines, as seen in our patient.

With regard to our patient, she was maintained on flurazepam for 44 years with no problems or side effects reported. She developed depression with prominent vegetative symptoms and psychomotor agitation following the discontinuation of flurazepam. These symptoms were not associated with physical symptoms suggesting benzodiazepine withdrawal, such as tremors, confusion, or sweating. In addition, these symptoms did not respond to the use of both temazepam and nitrazepam, as would be expected in cases of benzodiazepine withdrawal. Our patient's depressive symptoms worsened with the use of antidepressants, and she developed psychotic symptoms following the use of various antidepressants (mirtazapine, escitalopram, and venlafaxine), aligning with the agitated depression described by Koukopoulos et al. [1]. However, her symptoms improved with the use of other benzodiazepines, such as the combined use of alprazolam and zopiclone, in addition to the use of olanzapine. Several reports suggested that the antidepressant effect of benzodiazepines is seen mainly with triazolobenzodiazepine [2], and the role of antipsychotics for agitated depression was suggested by Koukopoulos et al. [1].

Our case highlights the importance of the diagnosis of agitated depression or melancholia should be considered in any patient presenting with major depression with psychomotor agitation or retardation, sleep and appetite disturbances, fixated thought process, and difficulty making decisions. Our patient recognized that her symptoms worsened with the use of various antidepressants and improved later on a combination of olanzapine, alprazolam, zopiclone, and venlafaxine. It is essential to consider the possibility of dementia or significant problems like brain metastasis in older patients who exhibit similar symptoms. However, the sudden onset of symptoms, their connection to stopping flurazepam, deterioration when using different antidepressants, and their complete resolution with successful treatment all suggest a diagnosis of agitated depression. This case has limitations, such as the lack of systematized symptom measurement based on validated clinical scales.

In conclusion, misrecognition of agitated depression can result in the problematic trials of several antidepressants, which can be associated with the worsening and emergence of suicidal ideations and psychotic symptoms. There is paucity in the literature about the treatment of agitated depression. Despite the limitations of our results, they are based on a single case report and previous small-scale studies; agitated depression deserves more focus as some options of treatments commonly prescribed for depression can potentially result in significant worsening of symptoms and adverse outcomes.

## Figures and Tables

**Figure 1:**
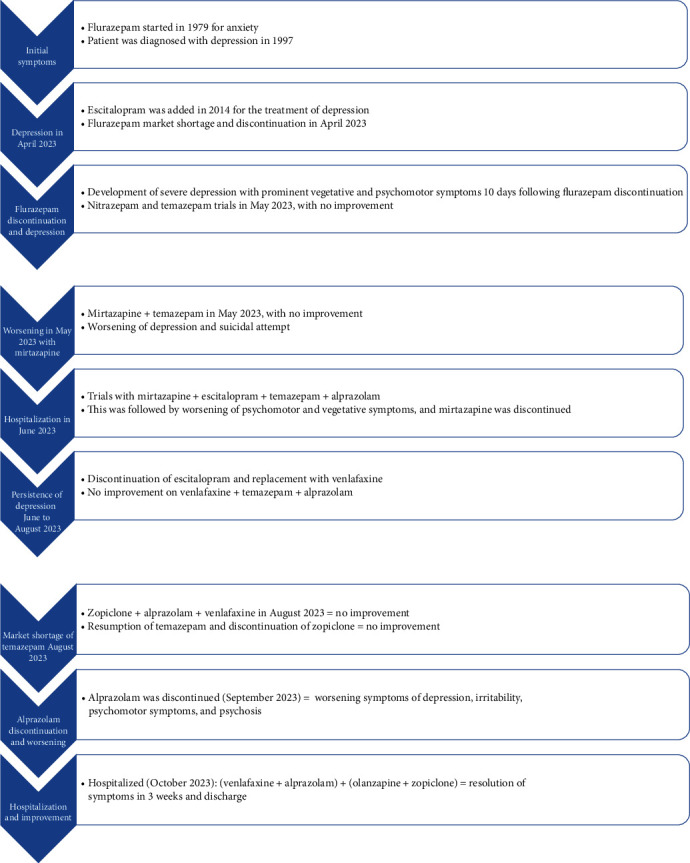
Summary of depressive disorder course following flurazepam discontinuation.

## Data Availability

Data sharing does not apply to this article as no datasets were generated or analyzed during the current study.
